# A rare case of terminal ileum diverticulosis in a-32-year old woman

**DOI:** 10.1016/j.amsu.2022.104106

**Published:** 2022-06-28

**Authors:** Zainab Zeino, Najah Zeno, Lina Kour Nassan, Wael Alkhaleel, Ahmad Ghazal

**Affiliations:** a Faculty of Medicine, University of Aleppo, Aleppo, Syria; b Department of General Surgery, University of Aleppo Hospital, Aleppo, Syria

**Keywords:** Terminal ileum diverticulosis, Perforated diverticula, Case report

## Abstract

**Introduction:**

and importance: Acquired small bowel diverticulosis is rare 1% and the ileum is the less frequent occurring site 15%. Most cases were multiple and asymptomatic. This is the first case of perforated terminal ileum diverticula in a 32-year-old woman and successfully managed surgically.

**Case presentation:**

We describe a rare case of terminal ileal diverticulosis, one of the diverticula was perforated, in a 32-year-old female who presented with a 2-days history of sudden epigastric and periumbilical pain and had been managing surgically. At surgery, we found multiple diverticula, one of them was perforated. Microscopically, the specimen confirmed that the diagnosis was diverticulosis.

**Conclusion:**

Diverticulosis should be included in the differential diagnosis of a sudden epigastric and periumbilical pain even in this age.

## Highlights

• Small bowel diverticulosis is not common comparing with colonic diverticulosis and occurs in up to 1% of patients.
• Diverticula is more common in males than females.
• We report a case of terminal ileum diverticulosis and a perforation of one diverticulum.

## 1 Introduction

Small intestine diverticula are the herniation of the mucosa and submucosa, through the muscle layer [1]. Acquired small bowel diverticulosis is not common comparing with colonic diverticulosis and occurs in up to 1% of patients. It mostly affects jejunum (80%) and the ileum is the less frequent occurring site (15%) [2,3].

The majority of cases are located on the mesenteric border and are often multiple and asymptomatic except in cases with complications such as perforation, inflammation and hemorrhage [3].

We present a case of a 32- year-old woman who has a terminal ileum diverticulosis and a perforation of one diverticulum diagnosed by pathology report and managed successfully. In our knowledge, this is an unusual clinical scenario encountered in clinical practice.

This case report has been reported in accordance with the SCARE criteria [9].

## 2 Case presentation

A 32-year-old woman presented to the emergency department complaining of a sudden epigastric and periumbilical pain responds to analgesics with vomiting, fever and constipation during the last two days.

A detailed surgical history revealed that the patient underwent a hemi-resection of medullary carcinoma of thyroid which led to hypothyroid later, as well as she also underwent a resection of leiomyomas uteri two years ago. In addition, she had some cardiac arrhythmias. But she had no significant allergic or familial history.

**Fig. 1:**
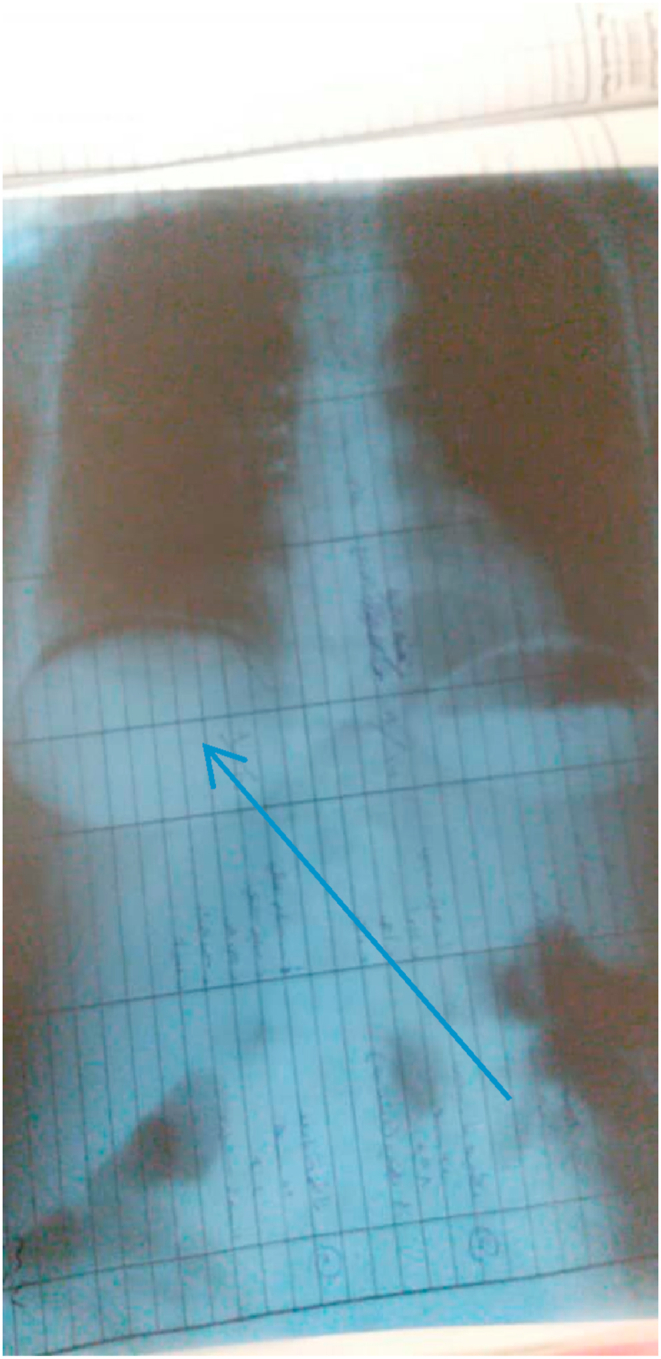
Preoperative X-ray images. Free air under the diaphragm which referred to perforation.

Vital signs were normal and the chest examination was also normal,but her abdominal examination confirmed a rigid abdomen with general tenderness.All her lab tests were within normal ranges except left shift and mild anemia (hemoglobin 9.6 g/dl, hematocrit 28.6%, MCV = 69.8 fl). Chest plain radiograph showed free air under the diaphragm which referred to perforation (Fig. 1).

**Fig. 2:**
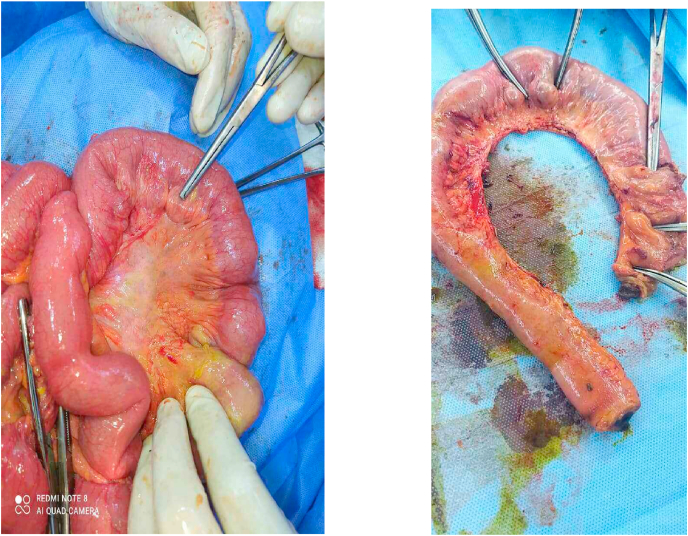
Intraoperative findings. Nine diverticular herniation through muscular wall, one of them was perforated.

In result of acute abdomen condition and perforation which was indicated by radiographic findings, the surgical decision was taken to do an exploratory laparoscopy as a surgical abdomen intervention. Surprisingly, we found a loop of ileum with a diameter of 40 cm, containing of multiple diverticula along the mesenteric border and one of them was perforated which was a 20 cm away from the ileocecal conjunction (Fig. 2). And the rest of the gastrointestinal tract was normal. The loop was resected and an ileal-ileal anastomosis was fashioned.

**Fig. 3:**
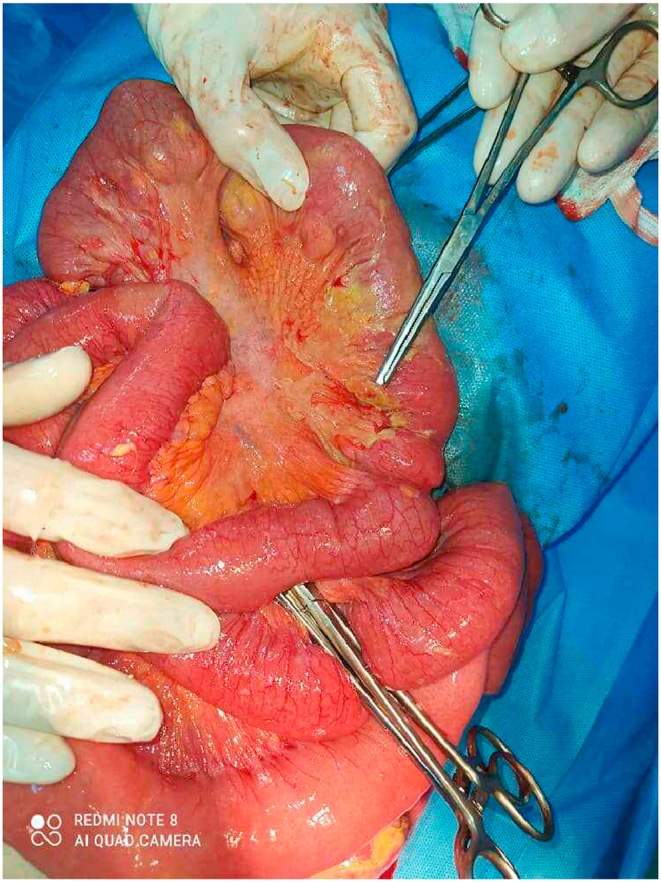
Intraoperative image showing the site of ileal diverticular perforation on the mesenteric border.

The pathology report showed normal intestine mucosa, marked edema of intestinal wall with presence of 9 diverticular herniation through muscular wall, one of them was perforated (Fig. 3), with no malignant structures in received specimen. The final diagnosis of excisional biopsy was diverticulosis and presence of one reactive lymph node.

Consequently, she was discharged home on the third post-operative day with six months of follow-up which demonstrated a good result and her state was improved.

## 3 Discussion

Diverticulosis is a disease characterized by the presence of one or more small, bulging pouches which form in the lining of particular structures in the gastrointestinal tract [1]. The diverticula, otherwise called the herniation through the muscle layer consisted both of mucosa and submucosa layers [4].

Diverticula is more common in males than females, with an approximate ratio of 2:1. In addition, the age wise distribution of diverticulosis increases in the elderly patients especially from (60s–70s) [2].

Non-Meckel small bowel diverticulosis is not common comparing with colonic diverticulosis and occurs in approximately 1% of the population. It mostly affects the jejunum (80%), while the ileum (15%) and (5%) in both jejunum and ileum [2,3,5].

Terminal ileum diverticula is an extremely rare disease that may lead to an acute abdomen mimicking appendicitis, as well as the mesenteric location is the most common, which are similar to our case [3,5].

Few cases were reported surprisingly talking about diagnosis of multiple diverticula in terminal ileum including our study. Our case is one of the youngest reported and occurs in a 32-year-old female.

Most diverticula of the small bowel are silent and asymptomatic and are discovered incidentally during imaging studies or in the operation room as in our case [2]. The first screening method we used was X-ray because its inexpensive and easily accessible which was adequate to initiate the surgical intervention to manage the perforation.

Ramzee et al. reported that diverticulosis is mostly caused by obesity, smoking, elderly age, drugs, diet and sedentary lifestyle [6], but it is worth noting that our patient do not have any of these reasons we mentioned above.

The complications occur in approximately 10% which include bleeding, perforation and diverticulitis resulting from bacterial infection. Particularly, the presence of complication makes the condition symptomatic [7].

We shared a perforated ileal diverticula which is not common case. However, Rajaguru et al. observed that patients with ileal diverticulosis are more likely to develop complications, such as perforation, than jejunal and duodenal diverticulosis [2].

Jeong et al. emphasized that in asymptomatic presentation of cases, we used conservative management which is adequate. Instead, in symptomatic cases, surgical resection is often the treatment of choice, something that was done in our case.

According to Saijo et al. who presented four elderly cases of perforation of terminal ileum diverticulosis which are managed surgically except one case was managed with conservative therapy; the high mortality is due to delayed diagnosis and elderly age because of the perforated diverticulum which is highly caused death, ranging from 21% to 40 [3,8]. The post-operative state of our patient was good and she made a full recovery.

The combination of young age, rarity of anatomical location and complication, and efficiently managed her symptoms had made our case unique.

## 4 Conclusion

In this study, we evaluated the outcomes of surgical management of terminal ileum diverticulosis with perforated diverticulum which need to have in mind despite the rarity of the case. Although this condition is not a disease that surgeons often see in clinical practice, but they need to be cautious about recommending a surgery for those adults patients.

The differential diagnosis of the surgical abdomen have a broad horizon and the medicine keep adding something new.

## Ethical approval

Not applicable.

## Sources of funding

This research was not funded.

## Author contribution

AG, WA performed the current surgery, supervised the study, drafted the article, analyzed and interpreted the data, and critically revised the article. ZZ, NZ, LK made a substantial contribution to conception and design, acquisition of data, analysis, and interpretation of data, drafting, and revision of the article. All authors read and approved the final version of the manuscript.

## Registration of research studies

1. Name of the registry:
2. Unique identifying number or registration ID:
3. Hyperlink to your specific registration (must be publicly accessible and will be checked):

## Guarantor

Dr. Ahmad Ghazal.

## Consent

A written informed consent was obtained from the patient for publication of this case report and accompanying images.

## Provenance and peer review

Not commissioned, externally peer-reviewed.

## Declaration of competing interest

The authors declare no conflict of interest.
