# Supplementary material for: A rare case of terminal ileum diverticulosis in a-32-year old woman
Source: Ann Med Surg (Lond). 2022 Jun 28;80:104106. doi: 10.1016/j.amsu.2022.104106 (PMC9283505; doi:10.1016/j.amsu.2022.104106)
Supplement: Multimedia component 1 [file mmc1.docx]

| **SCARE Checklist** | | | |
| --- | --- | --- | --- |
| **Topic** | **Item** | **Checklist item description** | **Page Number** |
| **Title** | **1** | **A rare case of terminal ileum diverticulosis in a-32-year old woman.** | 1 |
| **Key Words** | **2** | Terminal ileum diverticulosis; perforated diverticula; case report. | 1 |
| **Abstract** | **3a** | Acquired small bowel diverticulosis is rare 1% and the ileum is the less frequent occurring site 15%. Most cases were multiple and asymptomatic. This is the first case of perforated terminal ileum diverticula in a 32-year-old woman and successfully managed surgically. | 1 |
|  | **3b** | We describe a rare case of 32-year-old female who presented with a 2-days history of sudden epigastric and periumbilical pain. At surgery, we found multiple diverticula, one of them was perforated. |  |
|  | **3c** | The final diagnosis was terminal ileal diverticulosis, one of the diverticula was perforated. and we had been managing this case surgically. |  |
|  | **3d** | Diverticulosis should be included in the differential diagnosis of a sudden epigastric and periumbilical pain even in this age. |  |
| **Introduction** | **4** | Small intestine diverticula are the herniation of the mucosa and submucosa, through the muscle layer. (1) Acquired small bowel diverticulosis is not common comparing with colonic diverticulosis and occurs in up to 1% of patients. It mostly affects jejunum (80%) and the ileum is the less frequent occurring site (15%). (2, 3)  The majority of cases are located on the mesenteric border and are often multiple and asymptomatic except in cases with complications such as perforation, inflammation and hemorrhage. (3)  In our knowledge, this is an unusual clinical scenario encountered in clinical practice.  This case report has been reported in accordance with the SCARE criteria. | 1 |
| **Patient Information** | **5a** | A 32-year-old woman, | **1** |
|  | **5b** | presented to the emergency department complaining of a sudden epigastric and periumbilical pain responds to analgesics with vomiting, fever and constipation during the last two days. |  |
|  | **5c** | A detailed surgical history revealed that the patient underwent a hemi-resection of medullary carcinoma of thyroid which led to hypothyroid later, as well as she also underwent a resection of leiomyomas uteri two years ago. In addition, she had some cardiac arrhythmias. |  |
|  | **5d** | But she had no significant allergic or familial history. |  |
| **Clinical Findings** | **6** | Abdominal examination confirmed a rigid abdomen with general tenderness. Her chest and vital signs were normal.  All her lab tests were within normal ranges except left shift and mild anemia (hemoglobin 9.6 g/dl, hematocrit 28.6%, MCV=69.8 fl). Chest plain radiograph showed free air under the diaphragm which referred to perforation. (Figure 1) | 2 |
| **Timeline** | **7** | A 32-year-old woman presented to the emergency department complaining of a sudden epigastric and periumbilical pain responds to analgesics with vomiting, fever and constipation during the last two days.  A detailed surgical history revealed that the patient underwent a hemi-resection of medullary carcinoma of thyroid which led to hypothyroid later, as well as she also underwent a resection of leiomyomas uteri two years ago. In addition, she had some cardiac arrhythmias. But she had no significant allergic or familial history.  Abdominal examination confirmed a rigid abdomen with general tenderness. Her chest and vital signs were normal.  All her lab tests were within normal ranges except left shift and mild anemia (hemoglobin 9.6 g/dl, hematocrit 28.6%, MCV=69.8 fl). Chest plain radiograph showed free air under the diaphragm which referred to perforation (Figure 1). | 2 |
| **Diagnostic Assessment** | **8a** | Abdominal examination confirmed a rigid abdomen with general tenderness. Her chest and vital signs were normal.  All her lab tests were within normal ranges except left shift and mild anemia (hemoglobin 9.6 g/dl, hematocrit 28.6%, MCV=69.8 fl). Chest plain radiograph showed free air under the diaphragm which referred to perforation (Figure 1). | 2 + 3 |
|  | **8b** | There was no impediment or diagnostic challenges. |  |
|  | **8c** | In result of acute abdomen condition and perforation which was indicated by radiographic findings, the surgical decision was taken to do an exploratory laparoscopy as a surgical abdomen intervention. |  |
|  | **8d** | Surprisingly, we found a loop of ileum with a diameter of 40 cm, containing of multiple diverticula along the mesenteric border and one of them was perforated which was a 20 cm away from the ileocecal conjunction (Figure 2). And the rest of the gastrointestinal tract was normal. The loop was resected and an ileal-ileal anastomosis was fashioned.  The pathology report showed normal intestine mucosa, marked edema of intestinal wall with presence of 9 diverticular herniation through muscular wall, one of them was perforated (Figure 3), with no malignant structures in received specimen. The final diagnosis of excisional biopsy was diverticulosis and presence of one reactive lymph node. |  |
| **Therapeutic Intervention** | **9a** | The patient did not tell any previous illnesses. | 2+3 |
|  | **9b** | Not mentioned |  |
|  | **9c** | Not mentioned |  |
|  | **9d** | A general surgeons. |  |
|  | **9e** | Surprisingly, we found a loop of ileum with a diameter of 40 cm, containing of multiple diverticula along the mesenteric border and one of them was perforated which was a 20 cm away from the ileocecal conjunction (Figure 2). And the rest of the gastrointestinal tract was normal. The loop was resected and an ileal-ileal anastomosis was fashioned.  The pathology report showed normal intestine mucosa, marked edema of intestinal wall with presence of 9 diverticular herniation through muscular wall, one of them was perforated (Figure 3), with no malignant structures in received specimen. The final diagnosis of excisional biopsy was diverticulosis and presence of one reactive lymph node. |  |
|  | **9f** | Not mentioned |  |
| **Follow-up and**  **Outcomes** | **10a** | Consequently, she was discharged home on the third post-operative day with six months of follow-up which demonstrated a good result and her state was improved. | 2+3 |
|  | **10b** | Not mentioned |  |
|  | **10c** | Not mentioned |  |
|  | **10d** | Chest plain radiograph showed free air under the diaphragm which referred to perforation (Figure 1).  In result of acute abdomen condition and perforation which was indicated by radiographic findings, the surgical decision was taken to do an exploratory laparoscopy as a surgical abdomen intervention. Surprisingly, we found a loop of ileum with a diameter of 40 cm, containing of multiple diverticula along the mesenteric border and one of them was perforated which was a 20 cm away from the ileocecal conjunction (Figure 2). And the rest of the gastrointestinal tract was normal. The loop was resected and an ileal-ileal anastomosis was fashioned.  The pathology report showed normal intestine mucosa, marked edema of intestinal wall with presence of 9 diverticular herniation through muscular wall, one of them was perforated (Figure 3), with no malignant structures in received specimen. The final diagnosis of excisional biopsy was diverticulosis and presence of one reactive lymph node. |  |
| **Discussion** | **11a** | What distinguishes our case amd makes it unique that the combination of young age, rarity of anatomical location and complication, and efficiently managed her symptoms. | 4 + 5 |
|  | **11b** | (Most diverticula of the small bowel are silent and asymptomatic and are discovered incidentally during imaging studies or in the operation room as in our case. (2) )  (Ramzee et al reported that diverticulosis is mostly caused by obesity, smoking, elderly age, drugs, diet and sedentary lifestyle (6), but it is worth noting that our patient do not have any of these reasons we mentioned above.)  (The complications occur in approximately 10% which include bleeding, perforation and diverticulitis resulting from bacterial infection. Particularly, the presence of complication makes the condition symptomatic. (7) )  (Jeong et al emphasized that in asymptomatic presentation of cases, we used conservative management which is adequate. Instead, in symptomatic cases, surgical resection is often the treatment of choice, something that was done in our case.)  (According to Saijo et al. who presented four elderly cases of perforation of terminal ileum diverticulosis which are managed surgically except one case was managed with conservative therapy; the high mortality is due to delayed diagnosis and elderly age because of the perforated diverticulum which is highly caused death, ranging from 21% to 40 (3, 8) ) |  |
|  | **11c** | All the sections of discussion. |  |
|  | **11d** | Terminal ileum diverticulosis with perforated diverticulum need to have in mind despite the rarity of the case. Although this condition is not a disease that surgeons often see in clinical practice, but they need to be cautious about recommending a surgery for those adults patients.  The differential diagnosis of the surgical abdomen have a broad horizon and the medicine keep adding something new. |  |
| **Patient Perspective** | **12** | The post-operative state of our patient was good and she made a full recovery. | 5 |
| **Informed Consent** | **13** | Consent was taken from the patient. | 5 |
| **Additional Information** | **14** | No conflicts of Interest and there are no sources of funding. | 5+6 |
